# Procalcitonin as a biomarker of bacterial infection in critically ill patients admitted with suspected Sepsis in Intensive Care Unit of a tertiary care hospital

**DOI:** 10.12669/pjms.37.7.4183

**Published:** 2021

**Authors:** Afshan Bibi, Nida Basharat, Muhammad Aamir, Zujaja Hina Haroon

**Affiliations:** 1 Afshan Bibi (FCPS Chemical Pathology), Armed Forces Institute of Pathology (AFIP), Rawalpindi, Pakistan; 2 Nida Basharat (FCPS Chemical Pathology), Armed Forces Institute of Pathology (AFIP), Rawalpindi, Pakistan; 3 Muhammad Aamir (FCPS Chemical Pathology), Armed Forces Institute of Pathology (AFIP), Rawalpindi, Pakistan; 4 Zujaja Hina Haroon (FCPS Chemical Pathology) Armed Forces Institute of Pathology (AFIP), Rawalpindi, Pakistan

**Keywords:** Biomarker, Blood culture, Critically ill, Procalcitonin, Sepsis

## Abstract

**Objective:**

To compare the diagnostic accuracy of procalcitonin (PCT), C- reactive protein (CRP), total leukocyte count (TLC) and lactate in critically ill patients admitted with suspicion of sepsis.

**Methods:**

It was a cross sectional study conducted at the department of Chemical Pathology and Endocrinology AFIP, Rawalpindi, in collaboration with Medical and surgical intensive care units (ICU) of CMH Rawalpindi from January 2019 to December 2019. A total of 126 patients of both genders with age above 18 years and fulfilling the inclusion criteria of systemic inflammatory response syndrome (SIRS) were inducted in the study.

**Results:**

Out of 126 patients 82 (65%) patients have positive blood culture results. Male predominance was noted in patients with positive blood culture. Out of 82 patients with positive blood culture results 69(84%) patients have positive PCT results as well whereas 13(15%) patients with positive blood culture results have negative PCT values. 57(69%) patients had Gram negative bacterial infection and 25(30%) patients had Gram positive bacterial infection. Significant difference was noted between the medians of PCT in blood culture positive and blood culture negative group (p value< 0.05) whereas no significant difference was found between medians of CRP, TLC and lactate between blood culture positive and blood culture negative patients (p value > 0.05). ROC curve analysis of PCT, CRP and TLC were done, keeping blood culture as reference standard, PCT showed largest area under the curve (AUC) and clearly outperformed TLC and CRP. PCT showed AUC of 0.781 as compared to CRP and TLC, which was 0.568 and 0.617 respectively. PCT showed sensitivity of 93.9%, specificity of 47.7%, positive predictive value (PPV) of 77% and negative predictive value (NPV) of 80.8%.

**Conclusion:**

Higher NPV makes it a reliable marker for screening out sepsis in suspected cases.

## INTRODUCTION

Worldwide one in five deaths occur due to sepsis, 11 million people die every year with sepsis.^1^ Sepsis does not discern among age, gender, social status or geographic boundaries. In 2017, almost half of the sepsis cases worldwide occurred in children resulting in 2.9 million deaths in children under five years of age.^1^ Almost 60-80% of deaths are caused by sepsis in developing countries like Pakistan. Prevalence of sepsis in critically ill patients admitted to ICU was estimated to be around 28.3% in Pakistan.^2^

Sepsis is life-threatening condition occurring due to dysregulated host response to infection which may lead to multi-organ dysfunction and eventually death.^3^ Sepsis ranges in severity from systemic inflammatory response syndrome to septic shock. Epidemiological data show that mortality is 25–30% higher in patients with sepsis and this excess mortality is result of delayed diagnosis despite improvement in antimicrobial therapy.^4^ Timely and appropriate clinical decision-making is of utmost importance in cases of suspected sepsis.

Although a multitude of markers like CRP, TLC, erythrocyte sedimentation rate (ESR), lipopolysaccharide (LPS) binding protein have been proposed in the field of sepsis, but the most validated remains procalcitonin.^5^ Various studies conducted over last decade revealed its importance as predictive marker for bacterial infections.^6^ In addition, its methodology is less time consuming and labor intensive and provides prompt results for early start of treatment. PCT values are directly related to severity and outcome of sepsis.^7^ PCT levels > 0.5 ng/ml are highly suggestive of antibiotic requirement.^8^

Procalcitonin, precursor of calcitonin, is synthesized in C cells of thyroid gland. Bacterial toxin mediated production of inflammatory cytokines stimulates other organs like kidneys, liver, lungs, fat and muscles to release PCT into bloodstream.^9^ However, PCT is not released in viral infections as increased concentration of interferon γ in viral infections suppresses secretion of procalcitinin.^10^ PCT levels rise early in course of infection, remain high for 8-24 hours and then return to normal earlier than CRP.^7^ PCT has highest sensitivity and specificity for predicting systemic bacterial infection in adults. PCT is a sensitive marker for diagnosing neonatal infectious diseases as well.^11^ Furthermore PCT levels correlate well with severity of bacterial infection and bacterial load.^12^ Some studies in recent past demonstrated significantly higher PCT values in Gram negative bacteremia as compared to Gram positive bacteremia.^13^

Local data regarding diagnosis and outcome of sepsis is sparse. There is need of judicial use of antibiotics before arrival of blood culture results for prompt treatment of patients with sepsis. Therefore, present study was conducted to compare performance of procalcitonin, CRP, TLC and lactate compared to blood culture in critically ill patients admitted with suspicion of sepsis.

## METHODS

It was a cross sectional study conducted at Department of Chemical Pathology and Endocrinology AFIP, Rawalpindi, in collaboration with Medical ICU of CMH Rawalpindi from January 2019 to December 2019. Sample size calculated was 122 by WHO sample size calculator with 95% confidence interval, prevalence 28.3%^3^ and precision 8%, 126 patients were included in the study. Study was approved by Institutional Review Board of AFIP (IRB no. FC-CHP 14-1/READ-IRB/18/1058).

All patients fulfilling criteria for SIRS^14^ i.e.; temperature >100.4F or <96.8F, heart rate > 90 beats/min, respiratory rate > 20 breaths/min or PaCO_2_ < 32mmHg and WBC count >12000 cells/mm^3^ or <4000 cells/mm^3^ were included in study, while patients with chronic diseases comprising organ failure, all pregnant ladies and patients on prior antibiotic therapy were excluded from study. At time of admission to intensive care unit, signs and symptoms, clinical and laboratory data were collected. Samples were taken in plain gel tube for serum procalcitonin and CRP, potassium ethylenediamine tetraacetic acid (EDTA) tube for blood complete picture (TLC) and lactate and culture bottle for blood culture before administration of antibiotic therapy. Serum procalcitonin was analyzed on Abbott Architect i1000SR, serum CRP and lactate were analyzed on Siemens Advia 1800 and TLC on Sysmex haematology analyzer. Samples for blood culture were analyzed in suitable culture media on Bactec. Final confirmation of bacterial growth was made after incubation for seven days.

**Table I:** Baseline Characteristics of Study Population (Total Number, N = 126).

*Characteristics*	*Median (IQR)*
Age (years)	42 (33)
Temperature (F)	101( 5)
Heart Rate (beats/minute)	92 (15)
Respiratory Rate (breaths/min)	35(25)
Serum Pro calcitonin (ng/ml)	3.1 (12.5)
Serum C-Reactive Protein (mg/L)	56 (41.8)
Total Leukocyte Count	13,500 (3000)
Plasma Lactate (mmol/L)	1.5 (1.1)

**Table II:** Comparison of medians of study parameters in blood culture positive and negative group.

*Biomarker*	*Culture Positive Group (n= 82) Median (IQR)*	*Culture Negative Group (n=44) Median (IQR)*	*Reference Values of tests*	*p-Value*
PCT	5.6 (23.8)	0.35 (3.18)	<0.5ng/ml	<0.001
CRP	56 (38.5)	54 (39.7)	<10mg/l	0.21
TLC	14,000 (2500)	13,000 (3500)	4-10x10^9^_/l_	0.57
Lactate	1.6 (1.1)	1.4 (0.9)	0.5-2.2mmol/l	0.402

Data was analyzed by SPSS version 24. Qualitative variables were expressed in percentages and quantitative variables were expressed as median and interquartile range (IQR) as the data was non-parametric. Receiver operating characteristic (ROC) curve was applied for diagnostic accuracy.

## RESULTS

A total of 126 patients of both genders with age above 18 years fulfilling inclusion criteria were inducted in study. Out of 126 patients 92 (73%) were male and 34 (27%) were female. Shapirowilk test for normality showed non parametric distribution for all continuous variables (p<0.05). PCT value of >0.5ng/ml was taken positive for infection. Out of 126 patients 90(71%) patients have procalcitonin levels >0.5ng/ml and 82 (65%) patients have positive blood culture results. Sixty (73%) patients with positive blood culture were males. Out of 82 patients with positive blood culture results 69(84%) patients have positive PCT results whereas 13(15%) patients with positive blood culture results have negative PCT values. Fifty-seven (69%) patients had gram negative bacteremia and 25(30%) patients had gram positive bacteremia.

**Fig.1:**
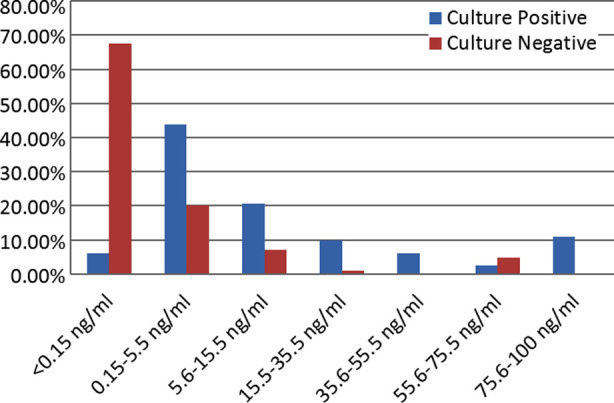
Procalcitinin values in blood culture positive and blood culture negative cases.

Medians of PCT, CRP, TLC and lactate were compared using Mann-Whitney U test, significant difference was noted between medians of PCT in culture positive and negative group. Majority of patients with negative blood culture had PCT levels below 0.15 ng/ml (Fig.1).

Medians of PCT in patients with gram positive and gram-negative bacterial infection were compared, but no significant difference was found (p-value = 0.274). Staphylococcus haemolyticus was the most common organism found on blood culture.

ROC curve analysis of PCT, CRP and TLC were done, keeping blood culture as reference standard, PCT showed largest AUC and clearly outperformed TLC and CRP. PCT showed AUC of 0.781 as compared to CRP, TLC and lactate which was 0.568 ,0.617 and 0.545 respectively.

**Fig.2:**
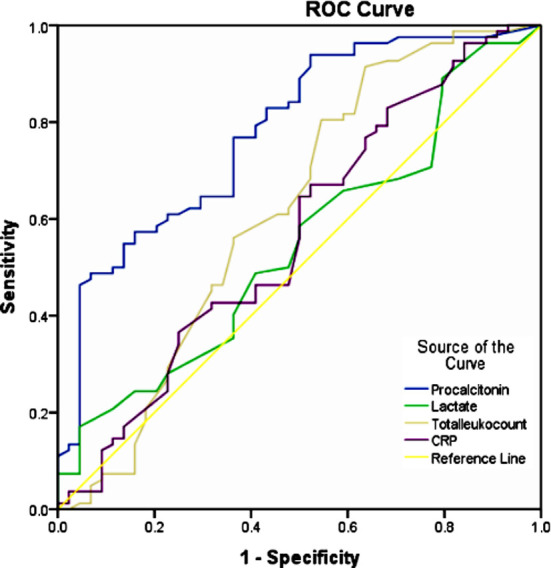
ROC Curve analysis, PCT, CRP, TLC and lactate.

PCT cutoff of 0.14-0.18ng/ml showed better diagnostic sensitivity, but with decreased specificity (48%). On other hand, a cutoff of 0.5 or 1.5ng/ml revealed better specificity (64%) at cost of sensitivity.

Further cross tabulation of blood culture and PCT results revealed sensitivity of 93.9%, specificity of 47.7%, PPV of 77% and NPV of 80.8%. Higher NPV makes it a reliable marker for screening out sepsis and avoiding unnecessary use of antibiotics.

## DISCUSSION

Early diagnosis and prompt antibiotic treatment are life savers in management of sepsis and results in reduced morbidity and mortality. PCT has become the most upcoming biomarker for the diagnosis of bacterial infection in last decade. PCT levels increase in bacterial infections from an extrathyroidal origin and rise early in course of infection, therefore making it useful biomarker for decision making regarding initiation of antibiotic therapy as results of blood culture are not available immediately.^15^ One hundred and twenty six blood specimens of critically ill patients admitted with suspicion of sepsis in intensive care unit were analyzed for PCT, CRP, TLC and lactate and compared with blood culture results as gold standard for bacterial infection. Majority of patients with positive blood culture were male (73%). Predominance of male gender in sepsis patients was in agreement with previous local and worldwide studies.^16^ Findings of current study revealed significantly higher PCT levels in patients with positive blood culture as compared to patients with negative blood culture (p<0.05). TLC, CRP and lactate levels were higher in blood culture positive patients but difference was not significant. CRP has been previously reported as nonspecific marker of infection in multiple studies.^17^ Lactate is widely used for early detection of sepsis, but it has proven more valuable in depicting prognosis of sepsis patients as raised lactate levels are more frequently associated with high mortality and organ dysfunction.^18^

In our study pro-calcitonin levels in patients with gram positive and gram-negative bacteremia were not significantly different. However higher PCT levels have been reported in patients with gram-negative infection than those with gram positive and fungal infection.^12^ We did not find significant difference in PCT levels in gram positive and gram-negative bacteremia probably because of small number of patients with positive blood culture results, secondly in Japan study PCT concentrations were significantly higher in patients who were infected with *E.coli*,^13^ whereas in our study population no patient was infected with E. Coli, thirdly we did not perform fungal culture.

On ROC curve analysis PCT clearly outperformed TLC and CRP for diagnosis of sepsis with AUC 0.78 (95%CI, 0.697- 0.865). In our study PCT has 93.9% sensitivity and 47.7% specificity in patients with suspected sepsis at cut off of 0.15ng/ml. Tan M et al reported in their meta analysis that PCT in terms of diagnostic accuracy has AUC of 0.85 (95% CI, 0.82-0.88), with a sensitivity and specificity of 0.80 (95% CI, 0.69-0.87) and 0.77 (95% CI, 0.60-0.88) respectively for diagnosis of sepsis.^19^ Ahmed S et al reported 93.8% sensitivity and 43.5% specificity at cut off of 0.5ng/ml. PCT had higher negative predictive value (81%) making it surrogate marker to rule out bacterial infection.^2^

PCT test also has its limitations, it is expensive compared to CRP and TLC. Although it is more specific for bacterial infections, it may falsely rise in cases of acute respiratory distress syndrome, chemical pneumonitis and severe falciparum malaria^20^ as we also observed in our study some of cases with positive PCT results did not show any growth on blood culture.

## CONCLUSION

Higher NPV makes serum procalcitonin levels a reliable marker for screening out sepsis in suspected cases.

## RECOMMENDATIONS

Serum PCT levels can be used as surrogate marker for excluding bacterial infection in suspected cases of septicemia. Implementing procalcitonin measurement in suspected patients can guide clinical decisions to diagnose bacterial infections earlier to reduce unnecessary tests, procedures, and length of hospital stay. This would result in reduction of mortality and total medical costs. We recommend further studies with larger sample size and serial measurement of PCT to better understand role and release kinetics of PCT in sepsis.

### Authors’ Contributions:

**AB** did manuscript writing, statistical analysis of data, takes the responsibility and is accountable for integrity and accuracy of all the aspects of work

**NB** did data collection.

**MA** did manuscript editing.

**ZH** conceived and designed the work.
